# A Note on the Supposed Cause of Milk-Sickness

**Published:** 1840

**Authors:** Charles W. Short


Art. XIII.—*Alleged causes of Milk-Sickness.* By C. W. Short, M. D.

A very intelligent farmer of Fayette county, Ky., who was frequently called to visit the country about Chillicothe, Ohio, where he owned a large tract of land, brought with him on one occasion the dried leaves of a plant, which he said was regarded in that quarter as the cause of that dreaded affection, the *milk-sickness*. On examination, these were ascertained to be the leaves of the *Caltha palustris*, a plant common throughout the middle and northern States, where it most frequently occurs in flat, wet lands, and hence is called "Marsh Marygold." In such situations, the yellow flowers of this plant appear early in the spring, before the leaves are fully developed; it then ripens its seeds and disappears by mid-summer. This plant belongs to the extensive tribe of *Ranunculæ*, the most of which are pungent and acrid; some are possessed of irritating, and others of narcotic, properties. In reference, therefore, to its botanical alliances, we are not inclined to deny that this vegetable might communicate deleterious properties to the flesh and milk of animals feeding upon it.

Another vegetable production, which is also frequently met with throughout the Western States, in poor hilly districts, has been suspected by some of the inhabitants of the "Eagle creek" and "Dry ridge" settlements in Kentucky, to be the cause of the same affection. This plant is the *Symphorea glomerata*, of botanists; and is a low shrub, found growing in dense patches, bearing small inconspicuous flowers, and a profusion of purple berries. It is of the family of *Caprifoliaceæ*—the honeysuckle tribe, none of which are poisonous or active.

*May*, 1840.

